# Frequency of Cognitive Impairment Among Malaysian Elderly Patients Following First Ischaemic Stroke—A Case Control Study

**DOI:** 10.3389/fpubh.2020.577940

**Published:** 2020-11-12

**Authors:** Zeena Mohamed Fuad, Hazlina Mahadzir, Syed Zulkifli Syed Zakaria, Norlinah Mohamed Ibrahim

**Affiliations:** ^1^Department of Medicine, Universiti Kebangsaan Medical Centre, Kuala Lumpur, Malaysia; ^2^Department of Paediatrics, Universiti Kebangsaan Medical Centre, Kuala Lumpur, Malaysia

**Keywords:** stroke, cognitive impairment, post-stroke dementia, post-stroke cognitive impairment, ischaemic stroke

## Abstract

**Background:** Stroke is highly prevalent globally and is an important cause of cognitive impairment and dementia.

**Aims:** We determined the frequency of post-stroke cognitive impairment (PSCI) at 1, 3, and 6 months among patients with first clinical ischemic stroke compared to risk and age-matched controls.

**Methods:** This study involved 32 cases and 32 controls, and was conducted over 6 months. Cases were inpatients aged >60 with first clinical ischemic stroke. Controls were age-matched subjects without prior stroke. Montreal Cognitive Assessment (MoCA) was performed in all patients at 1, 3, and 6 month post stroke. A MoCA score of <26 was used for mild PSCI and <22 for moderate PSCI (post stroke dementia).

**Results:** Post-stroke dementia was detected in 12 patients (37.5%) at 1^st^ month, in 13 (40.6%) at 3^rd^ month and 15 (48.4%) at 6^th^ months. Mild PSCI was present in 7 patients (21.6%) at 1 month, 16 patients (50%) at 3 months, and 15 patients (48%) at 6 months. The odds ratio (OR) for post-stroke dementia was 3.2 (95%CI 0.98–10.68; *p* = 0.05) at 1 month; 3.69(95% CI 1.13–12.11; *p* = 0.031) at 3 months, and 4.88 (95% CI 1.49–15.99; *p* = 0.009) at 6 months. Years of education was an independent predictor for dementia (OR 0.60; *p* = 0.046). The OR for post-stroke dementia at 6^th^ month was 7.23 with education level adjusted (95%CI 1.46–35.86, *p* = 0.015).

**Conclusion:** The frequency of PSCI was high as early as 1 month after stroke. Stroke alone conferred a 7.2 times risk for post-stroke dementia compared to controls.

## Introduction

Stroke is an important cause of cognitive impairment (1, 2). The Oxford Vascular Study, a large prospective study evaluating incidence of vascular events among participants living in Oxfordshire reported that the incidence dementia after a major stroke was 50 times higher than in the general population, in the year after a stroke (2). A systematic review on post stroke dementia yielded varying prevalence depending on the population studied and the number of strokes. A pooled prevalence of 7.4% (4.8–10) was obtained from population-based studies, whereas hospital-based studies had a higher prevalence of 41.3% (29.6–53.1) when recurrent strokes and pre-stroke dementia were not excluded (1).

The Malaysian National Stroke Registry had reported an increase in stroke incidence and prevalence from 2010 to 2014 most likely due to an increase in the prevalence of vascular risk factors (3). The Global Burden of Disease Study 2016 reported an increase in the burden of stroke worldwide, particularly in the low to middle income countries such as Malaysia (4). This increasing burden of stroke has a significant impact on the incidence and prevalence of post stroke cognitive impairment (PSCI) (5). Cognitive impairment and/ or dementia, when present is associated increased rates of institutionalization, mortality, and poor quality of life (6).

While there are some studies on the prevalence of dementia among Malaysians (7–9), data on post-stroke cognitive impairment is still lacking except for one study which evaluated the presence of post stroke cognitive impairment among stroke survivors in the community (10). Additionally, although cognitive impairment may become established from as early as 1-month post stroke, there are no national guidelines or recommendations on when to screen for cognitive impairment following an incident stroke. As there is increasing evidence for early cognitive rehabilitative interventions for improving cognition post stroke (11), it is therefore imperative to determine the incidence and prevalence of cognitive impairment after an incident stroke, and to understand the contributory risk factors so that treatment can be instituted appropriately.

This study was therefore conducted to determine the incidence and trajectory of cognitive impairment among patients with first-ever ischemic stroke compared to age-matched high risk controls over 6 months. We also determined independent risk factors which contributed toward the development of post-stroke dementia.

## Materials and Methods

### Study Design

This was a single-center prospective observational case-control study. Patients were recruited from the stroke unit, general medical wards, and from stroke registry of Hospital Canselor Tuanku Muhriz, Kuala Lumpur. Inclusion criteria for cases were: elderly patients aged ≥60 years, who were admitted with a first clinical ischaemic stroke to the medical wards or stroke care unit. Controls were age, gender and risk-factor matched, non-stroke patients attending the cardiology outpatient clinic or patients admitted to general medical wards, with a diagnosis other than stroke. Patients were excluded if they had previous strokes, a haemorrhagic stroke, diagnosis of dementia, or other neurodegenerative diseases such as Parkinson's disease, Alzheimer's disease. Ethics approval was obtained prior study conduct with an approval code. Informed consent was obtained prior to enrolment.

### Study Assessments

Patients were assessed by a single investigator. Baseline data which included demographics, level of education, and risk factors (hypertension, diabetes mellitus, dyslipidaemia, ischemic heart disease, and smoking) were collected into a semi-structured questionnaire. Patients and family members were questioned regarding any forgetfulness prior to the stroke to determine if there was a possibility of undiagnosed dementia or cognitive impairment.

Stroke was classified according to Oxfordshire Community project Classification (OCSP) as lacunar stroke (LaCI), posterior circulation stroke(PoCI), partial anterior circulation stroke(PACI), and total anterior circulation stroke (TACI) (12). The presence of white matter changes and old lacunar infarcts on computed tomography (CT) imaging were recorded.

The Montreal Cognitive Assessment (MoCA) was used to screen for the presence of cognitive impairment or dementia (13). A cut off score of <26 was used for mild cognitive impairment, a score of <22 was used for dementia (14, 15). All assessments were performed at 1, 3, and 6 months post-stroke by a single interviewer.

In the post-stroke patients, probable post stroke dementia was diagnosed using the NINDS AIREN criteria, which requires the presence of dementia by clinical history and examination, and documented by neuropsychological testing (MoCA <22); evidence of cerebrovascular disease by either history, clinical examination, or brain imaging; and the first 2 criteria to be reasonably related (16).

### Statistical Analysis

The sample size required for each arm, based on previous study (17) with study power set to 80% with 5% level of significance was 36. Statistical analyses were performed using IBM Statistical Package for Social Sciences (SPSS) version 21.0. Non-parametric tests were used as the data were not normally distributed. Categorical variables were analyzed using Fisher's exact test and Pearson Chi-square test where appropriate. The continuous variables were analyzed using Mann–Whitney *U*-test. Repeated Measurement Analysis using Friedman ANOVA test was done to determine change of MoCA over time between two groups. Kaplan-Meier Survival Analysis was done to estimate dementia free survival comparing two groups. Univariate followed by multivariate logistic regression was performed to determine the independent predictors for post-stroke dementia. Binary logistic regression analysis was performed to calculate the odds ratio for dementia between two groups of subjects. A *p* < 0.05 was considered statistically significant.

## Results

### Baseline Characteristics

A total of 64 subjects were finally recruited (32 stroke subjects and 32 control subjects). Of 41 patients with stroke initially recruited, only 32 completed the study. One patient died at home due to unknown cause and 8 patients withdrew from the study. One patient from the stroke group and one patient from the control group were unable to complete the 6^th^ month assessment. Both cases and controls were age and sex-matched. The median age among cases was 66.50 years (IQR 65.00–71.75) while among controls was 67.00 years (IQR 63.00–70.00). Baseline sociodemographic and the presence of underlying risk factors were comparable for cases and controls (Table 1).

**Table 1:** Baseline sociodemographic data of the subjects.

	**Cases (*n* = 32)**	**Control (*n* = 32)**	***p*-value**
Age (years)^***^			
Median (IQR)	66.50 (65.00–71.75)	67.00 (63.00–70.00)	0.803
**Sex** ***n*** (%)^*^			
Male	23 (35.9)	25 (39.1)	
Female	9 (14.1)	7 (10.9)	0.774
**Race** ***n*** (%)^**^			
Malay	9 (14.1)	10 (15.6)	
Chinese	20 (31.3)	21 (32.8)	
Indian	3 (4.7)	1 (1.6)	0.584
**Smoking** ***n*** (%)^*^			
Smoker	12 (18.8)	15 (23.4)	
Non-smoker	20 (31.3)	17 (26.6)	0.613
Education years^***^			
Median (IQR)	7 (3–9.25)	7 (3.5–10)	0.254
Hypertension *n* (%)^**^	30 (50)	30 (50)	1.00
DM *n* (%)^*^	14 (21.9)	19 (27.1)	0.317
Dyslipidemia *n* (%)^**^	29 (90.6)	30 (93.8)	0.641
IHD *n* (%)^**^	12 (18.8)	23 (35.9)	0.011

* *Analysis performed using Fisher's exact test*.

** *Analysis performed using Pearson Chi-Square test*.

*** *Analysis performed using Mann–Whitney U-test*.

*DM, Diabetes Mellitus; IHD, Ischemic Heart Disease*.

Among the stroke subjects 65% had lacunar stroke and 35% had non-lacunar stroke. As per OCSP classification, the majority of the patients had LaCI (59.4%), followed by PACI (21.9%) and PoCI (18.8%). None of the patients had a TACI perhaps since aphasic patients were excluded. In the stroke group, 50% had either white matter changes or old lacunar infarcts on the brain imaging.

### Post-stroke Dementia and MCI

At 1-month post stroke, 12 (37.5%) patients had PSD, while only 5 (15.6%) patients in the control group fulfilled the MoCA cut-off for dementia (*p* = 0.04). By the 3^rd^ month, 3 (40.6%) patients had developed dementia in the stroke group while the number remained unchanged in the control group (*p* = 0.025). By the 6^th^ month, a total of 15 (48.4%) had developed dementia in the stroke group, whereas the control group remained unchanged (Table 2).

**Table 2:** Frequency of PSCI (MCI and dementia) among stroke subjects and controls.

	**Stroke %**	**Controls %**	^*^P-value
**1st month**			
Normal	13 (40.6)	24(75.0)	
MCI	7 (21.6)	3 (9.4)	0.151
Dementia	12 (37.5)	5 (15.6)	0.044
**3**^rd^ **month**			
Normal	3 (9.4)	23 (71.9)	
MCI	16 (50.0)	4 (12.5)	0.003
Dementia	13 (40.6)	5 (15.6)	0.025
**6**^th^ **month**			
Normal	1 (3.2)	21 (65.6)	
MCI	15 (48.4)	6 (18.8)	0.031
Dementia	15 (48.4)	5 (15.6)	0.007

* *Fisher's exact test*.

*MCI, Mild Cognitive Impairment*.

At 1 month, 7 (21.9%) patients in the stroke group had mild PSCI (scored MoCA <26) while 3 (9.4%) patients in the control group had mild cognitive impairment (MCI) (*p* = 0.151). By the 3^rd^ month, 16 (50%) patients in the stroke group had developed mild PSCI (MoCA <26), while only 4 (12.5) in the control group had MCI (*p* = 0.003). At 6^th^ month, 15 (48.4%) patients had MCI in the stroke group, as one patient had progressed to dementia, while 2 new patients in the control group had developed MCI making a total of 6 (22.2%) (*p* = 0.031; Table 2).

### Risk Factors for Dementia

Years of education (*p*=0.020, OR 0.75, 95% CI 0.64-0.99) and non-lacunar stroke (*p*=0.024, OR 1.6, 95% CI 0.09-7.82) were significantly associated with dementia in the stroke group using univariate analysis (Table 3). The presence of diabetes, hypertension, dyslipidaemia, white matter changes and old lacunar infarcts on brain imaging were not significant risk factors for post-stroke dementia. Using multivariate regression, only years of education was found to be an independent predictor for developing post-stroke dementia (OR 0.60, 95%CI 0.36–0.99, *p* = 0.046; Table 4).

**Table 3:** Univariate logistic regression for risk factors for dementia at 6^th^ month.

	**OR**	**95% Confidence interval**	***p*-value**
Age	1.08	0.947–1.24	0.245
Sex (female)	0.6	0.119–3.03	0.537
Years of education	0.75	0.64–0.99	0.020
DM	1.07	0.256–4.49	0.925
Hba1C	1.21	0.80–1.84	0.373
Hypertension	6.18	0.26–146.7	0.260
IHD	0.98	0.227–4.25	0.981
Smoking	1.71	0.396–7.42	0.471
Depression	0.27	0.025–2.90	0.278
Type of stroke (non-lacunar)	1.6	0.09–7.82	0.024
Site of stroke (Left)	5.0	0.82–30.46	0.08
Presence of white matter changes or old lacunar infarct on CT brain	0.33	0.68–1.62	0.174
**OCSP classification**			
LaCI	0.90	0.129–0.16	0.908
PoCI	3.75	0.331–42.46	0.286

*DM, Diabetes Mellitus; IHD, Ischemic Heart Disease; OCSP, Oxfordshire Community Stroke Project; LaCI, Lacunar stroke; PoCI, Posterior circulation stroke; PACI, partial anterior circulation stroke*.

**Table 4:** Multivariate logistic regression for risk factors of dementia at 6^th^ month.

	**OR**	**95%Confidence interval**	***p*-value**
Age	1.22	0.85–1.76	0.281
Sex (female)	3.83	0.18–81.56	0.389
Years of education	0.60	0.36–0.99	0.046
DM	0.10	0.03–3.02	0.184
Dyslipidemia	8.56	0.009–7,974.42	0.538
IHD	1.98	0.13–29.79	0.621
Type of stroke (non-lacunar)	0.44	0.36–6.54	0.560
Presence of white matter changes or old lacunar infarct on CT brain	0.49	0.29–487.5	0.588

*DM, Diabetes Mellitus; IHD, Ischemic heart Disease*.

### Post-stroke Dementia Risk and Progression of Cognitive Impairment

Patients with stroke had significantly increased risk of developing cognitive impairment at 1 month compared to controls with an odds ratio (OR) of 3.2 (95%CI 05.98; *p* = 0.05). This increased to 3.69 at 3 months (95% CI 1.13–12.11; *p* = 0.031) and 4.88 at 6 months (CI 1.49–15.99; *p* = 0.009). After adjusting for educational level at 6^th^ month, the OR increased to 7.23 (95%CI 1.46–35.86, *p* = 0.015).

**Figure 1:**
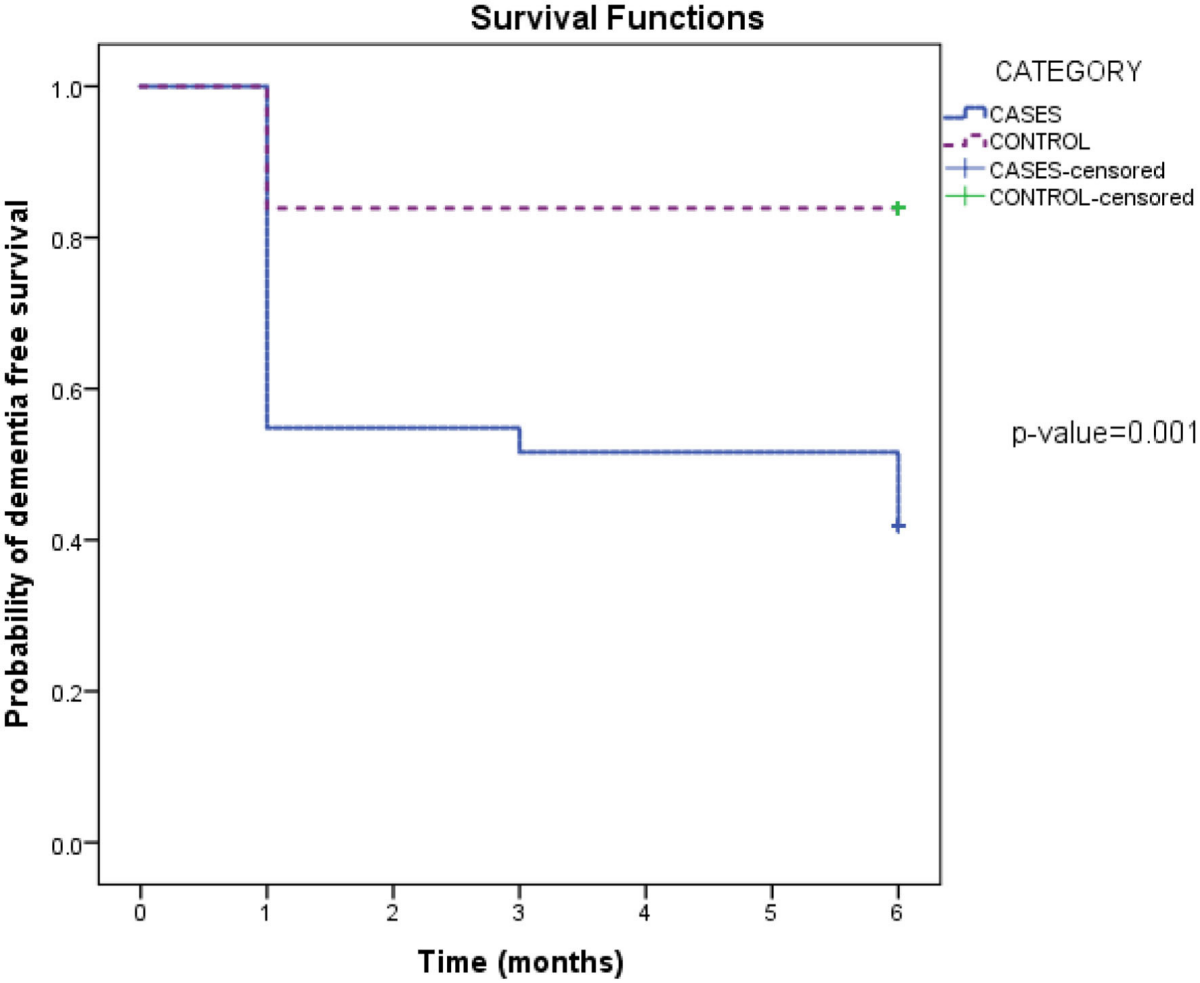
Estimate of survival free of dementia over 6 months between cases and controls.

Of the 7 patients with who had mild PSCI by MoCA score at 1 month, 3 progressed to post-stroke dementia at 6 months, and the rest remained as mild PSCI, with a conversion rate of 42.8%. None of the patients in the control group with MCI converted to dementia. A non-parametric repeated measurement analysis using Friedman ANOVA test to determine change over time for MoCA in 2 groups showed a greater rate of decline in the stroke group (chi-square 38.49, *p* < 0.001). Based on survival analysis, 50% of stroke patients remained dementia-free by the 6th month, whereas in the control group 85% were dementia free by the 6^th^ month. The mean estimate 6-month dementia-free survival was 3.6 in the test group and 5.2 in the control group, *p* = 0.007 (Figure 1).

## Discussion

Stroke leads to global cognition impairment in 44% of patients and impairment in individual domains in 30–35% of patients within 2–6 months of an index event (18). A recent meta-analysis of hospital-based studies which harmonized data on post stroke cognitive impairment (PSCI), assessed at ≥1 month, from stroke onset showed that the pooled prevalence of post stroke neurocognitive disorder (NCD) was 53.4%; with mild NCD (MCI) in 36.4% and major NCD (PSD) in 16.5% of patients (19). International guidelines have recommended screening for PSCI as early as 3–6 months, so that appropriate treatment can be instituted. In this study, we evaluated the frequency of post stroke mild cognitive impairment and moderate cognitive impairment at three time points (1, 3, and 6 month), to determine the trajectory of post stroke cognitive changes over time, and compared this against age, gender, and disease-matched controls.

As expected, our study showed a significantly higher prevalence of moderate PSCI (PSD) among our ischaemic stroke patients at all the three time points compared to age-matched controls. In addition, there was a steady rise in the prevalence of PSD over the 6-month period, from 37.5% at 1^st^ month, to 40.6% at the 3rd month, and to 48.4% at the 6th month. Similar to that observed with PSD, the prevalence mild PSCI among the stroke survivors was significantly higher compared to controls at 3 and 6 months. The prevalence of mild PSCI also increased steadily from 21.9% in the first month to 50% at 3 and 6 months. Taken together, the prevalence of cognitive impairment (mild and moderate) after an incident stroke in our study was 98.4%, which is alarmingly high. In contrast, a study, similar to ours, which screened for PSCI, at 2 and 6 months, in 325 subjects, using a MOCA cut off score of <26, reported that the prevalence of PSCI was highest at 2 months (66.4%), which declined slightly to 59.1% at 6 months (20). Most published studies have used single point assessments at either 3 or 6 months for the evaluation of dementia post stroke, thus yielding differing prevalence.

The progression of mild PSCI to PSD over the 6-month period was rather high in our study. Three out of 7 (42.8%) patients progressed from mild PSCI to PSD by the 6^th^ month. No changes in MoCA scores were observed in the control group. The risk of developing post stroke dementia at 6 months following a first clinical stroke was 7.2 times higher than that for someone of the same age, gender, educational level, and disease risks. This is perhaps one of the highest reported in the literature. On the other hand, a population-based study, involving 212 patients and 1,060 controls reported a 2.4 times risk of developing dementia after a first stroke (21).

Based on survival analysis, only 50% of stroke patients had MoCA scores ≥22 by the 6^th^ month, compared to 85% in the control group. Educational level was the only independent predictor for post stroke dementia from our study. Patients with longer years of education had significantly lower risk of developing post stroke dementia. Other risk factors including age, depression, type of stroke and the presence of white matter disease were not significantly associated with dementia in our study. In addition, the progression to moderate cognitive impairment was most significant in the first 3 months of stroke, although the MoCA scores continued to decline from 3 to 6-month post-stroke. Our findings are supported by another study which showed that although the probability of incident dementia declined over time following stroke, it was highest within the first 90 days following a stroke (22).

Studies on post stroke cognitive impairment in Malaysia are limited despite an increasing prevalence and mortality associated with stroke. To our knowledge, only one study had evaluated the prevalence of post-stroke cognitive impairment, and it was conducted in the community. In that study, 50 patients with single or recurrent strokes, attending day-care centers in Klang Valley, Malaysia were screened using the Addenbrooke's Cognitive Examination-Revised instrument (10). It was reported from that study that 76% of patients had post stroke cognitive impairment (10) Among the risk factors evaluated, only education level and age were found to be independent predictors of cognitive dysfunction in that study (10). Despite the methodological differences, the prevalence of PSD was high in our study and the above study; although in the latter, the prevalence was perhaps higher due to the inclusion of recurrent strokes. This could be partly due to the fact that post stroke rehabilitative management in Malaysia are primarily focused on improving physical mobility and speech with insufficient emphasis on cognitive rehabilitation therapy, which could have accounted for the high prevalence of PSD among Malaysians. Given the high progression of mild to PSD in our study, there is a pressing need to screen and treat cognitive impairment post stroke, as early as possible, to prevent progression.

There are several limitations in this study. Firstly, being a hospital-based study, we may have missed patients with mild stroke who did not seek medical attention. Secondly, we only included patients with ischaemic stroke and thus unable to determine the prevalence of PSCI in patients with haemorrhagic strokes. Thirdly, as this study was meant to determine the trajectory of cognitive decline, we did not evaluate the functional impairment associated with PSD, using the Barthel Index and hence unable to correlate the presence of PSD with functional activity. Finally, although MoCA is most valid and clinically feasible tool to screen for cognitive impairment post stroke (23), a cut off score of <26 for may have led to an overestimation of cases with PSCI. Finally, our sample size was relatively small compared to other studies. Nevertheless, findings from this study could be used to design larger clinical trials or studies to assess PSCI in the Malaysian population.

However, we believe our study has a number of strengths. This was the first study to report the trajectory of cognitive decline following an incident stroke among Malaysians. Being a prospective study, we were able to detect the development of new dementia among our patients more accurately. We had also utilized a lower MoCA cut score of <22 matched for years of education, for the diagnosis of dementia, to ensure that we did not overestimate the presence of dementia in our patients. This was based on previous validation studies conducted among Malaysians, and in Hong Kong, as both populations had similar years of education to our current cohort (14, 15). Additionally, our cases and controls were matched by age and sex as well as for other predisposing risk factors for vascular dementia, which allowed us to determine the true effect of the first stroke on the development of dementia in these patients.

In conclusion, our study showed that a first incident ischaemic stroke led to PSD in 48% of patients at 6 months, and conferred a 7.2 times risk for PSD compared to age- and risk-matched controls. These alarming rates strongly emphasize the need to institute cognitive rehabilitative strategies as part of stroke rehabilitative protocol in Malaysia. Future longitudinal studies, with longer follow up are warranted to understand the risks and burden of post-stroke dementia in our population, considering the increasing prevalence of stroke in Malaysia.

## Data Availability Statement

The raw data supporting the conclusions of this article will be made available by the authors, without undue reservation.

## Ethics Statement

The studies involving human participants were reviewed and approved by Secretariat of Research and Innovation, Faculty of Medicine, Universiti Kebangsaan Malaysia. Approval Code - FF-2016-435. The patients/participants provided their written informed consent to participate in this study.

## Author's Note

Apart from detrimental effects on functional abilities, stroke is a major contributing factor for dementia. Data on post-stroke cognitive impairment and dementia following first ischaemic stroke in Malaysia is lacking. Post-stroke cognitive impairment has a significant impact on overall prognosis independent of physical disability. This study showed that years of education was a significant factor for developing post-stroke dementia. These rates are quite alarming and suggest that patients with stroke should be screened and received early cognitive rehabilitation regardless of their baseline status given the high conversion rate to MCI and dementia over the 6-month period. Additionally, early treatment for those with MCI could potentially prevent progression to dementia.

## Author Contributions

ZMF contributed to writing proposal, data collection, analysis, and writing first draft. HM contributed to study conception and review of manuscript. SZSZ contributed to study design and statistical analysis. NMI contributed to initial study conception and design, data analysis, and critical review of the manuscript.

## Conflict of Interest

The authors declare that the research was conducted in the absence of any commercial or financial relationships that could be construed as a potential conflict of interest.

## References

[1] Pendlebury ST Rothwell PM. Prevalence, incidence, and factors associated with pre-stroke and post-stroke dementia: a systematic review and meta-analysis. Lancet Neurol. (2009) 8:1006–18. 10.1016/S1474-4422(09)70236-4 19782001

[2] Pendlebury ST Rothwell PM. Incidence and prevalence of dementia associated with transient ischaemic attack and stroke: analysis of the population-based oxford vascular study. Lancet Neurol. (2019) 18:248–58. 10.1016/S1474-4422(18)30442-3 30784556 PMC6390174

[3] Aziz ZA Lee YYL Ngah BA Sidek NN Looi I Hanip MR. Acute stroke registry malaysia, 2010-2014: results from the national neurology registry. J Stroke Cerebrovasc Dis. (2015) 24:2701–9. 10.1016/j.jstrokecerebrovasdis.2015.07.025 26338106

[4] Johnson CO Nguyen M Roth GA Nichols E Alam T Abate D Global, regional, and national burden of stroke, 1990–2016: a systematic analysis for the global burden of disease study 2016. Lancet Neurol. (2019) 18:439–58. 10.1016/S1474-4422(19)30034-1 30871944 PMC6494974

[5] Tatemichi T Paik M Bagiella E Desmond D Pirro M Hanzawa L. Dementia after stroke is a predictor of long-term survival. Stroke. (1994) 25:1915–9. 10.1161/01.STR.25.10.1915 8091433

[6] Rockwood K Wentzel C Hachinski V Hogan DB MacKnight C McDowell I. Prevalence and outcomes of vascular cognitive impairment. Neurology. (2000) 54:447. 10.1212/WNL.54.2.447 10668712

[7] Hamid TA Krishnaswamy S Abdullah SS Momtaz YA. Sociodemographic risk factors and correlates of dementia in older Malaysians. Dement Geriatr Cogn Disord. (2010) 30:533–9. 10.1159/000321672 21252548

[8] Kua EH Ko SM. Prevalence of dementia among elderly Chinese and Malay residents of Singapore. Int Psychogeriatri. (1995) 7:439–46. 10.1017/S1041610295002183 8821351

[9] Ali RA Mathews S Prevalence of dementia among elderly malays in an urban settlement in Malaysia. Neurol J SouthEast Asia. (1997). 2:154–62.

[10] Mohd Zulkifly MF Ghazali SE Che Din N Subramaniam P. The influence of demographic, clinical, psychological and functional determinants on post-stroke cognitive impairment at day care stroke center, Malaysia. Malays J Med Sci. (2016) 23:53–64. 10.1155/2016/3456943 27547115 PMC4976714

[11] Merriman NA Sexton E McCabe G Walsh ME Rohde D Gorman A. Addressing cognitive impairment following stroke: systematic review and meta-analysis of non-randomised controlled studies of psychological interventions. BMJ Open. (2019) 9:e024429. 10.1136/bmjopen-2018-024429 30819706 PMC6398645

[12] Pittock SJ Meldrum D Hardiman O Thornton J Brennan P Moroney JT. The oxfordshire community stroke project classification: correlation with imaging, associated complications, and prediction of outcome in acute ischemic stroke. J Stroke Cerebrovasc Dis. (2003) 12:1–7. 10.1053/jscd.2003.7 17903897

[13] Nasreddine ZS Phillips NA Bédirian V Charbonneau S Whitehead V Collin I The montreal cognitive assessment, MoCA: a brief screening tool for mild cognitive impairment. J Am Geriatr Soc. (2005) 53:695–9. 10.1111/j.1532-5415.2005.53221.x 15817019

[14] Yeung P Wong L Chan C Leung J Yung C. A validation study of the Hong Kong version of montreal cognitive assessment (HK-MoCA) in Chinese older adults in Hong Kong. Hong Kong Med J. (2014) 20:504–10. 10.12809/hkmj144219 25125421

[15] Cheah WK Teh HL Huang DXH Ch'ng ASH Choy MP Teh EE Validation of Malay version of montreal cognitive assessment in patients with cognitive impairment. Clin Med Res. (2014) 3:56–60. 10.11648/j.cmr.20140303.11

[16] Román GC Tatemichi TK Erkinjuntti T Cummings J Masdeu J Garcia Ja. Vascular dementia diagnostic criteria for research studies: report of the NINDS-AIREN international workshop. Neurology. (1993) 43:250. 10.1212/WNL.43.2.250 8094895

[17] Tatemichi TK Desmond D Mayeux R Paik M Stern Y Sano M. Dementia after stroke Baseline frequency, risks, and clinical features in a hospitalized cohort. Neurology. (1992) 42:1185. 10.1212/WNL.42.6.1185 1603346

[18] Censori B Manara O Agostinis C Camerlingo M Casto L Galavotti B. Dementia after first stroke. Stroke. (1996) 27:1205–10. 10.1161/01.STR.27.7.1205 8685929

[19] Barbay M Diouf M Roussel M Godefroy O. Systematic review and meta-analysis of prevalence in post-stroke neurocognitive disorders in hospital-based studies. Dement Geriatr Cogn Disord. (2018) 46:322–34. 10.1159/000492920 30504699

[20] Nijsse B Visser-Meily JMA Mierlo MLV Post MWM Kort PLMd Heugten CMV. Temporal evolution of poststroke cognitive impairment using the montreal cognitive assessment. Stroke. (2017) 48:98–104. 10.1161/STROKEAHA.116.014168 27899753

[21] Ivan CS Seshadri S Beiser A Au R Kase CS Kelly-Hayes M. Dementia after stroke the framingham study. Stroke. (2004) 35:1264–8. 10.1161/01.STR.0000127810.92616.78 15118167

[22] Tatemichi TK Foulkes MA Mohr J Hewitt JR Hier DB Price TR. Dementia in stroke survivors in the stroke data bank cohort. Prevalence, incidence, risk factors, and computed tomographic findings. Stroke. (1990) 21:858–66. 10.1161/01.STR.21.6.858 2349588

[23] Burton L Tyson SF. Screening for cognitive impairment after stroke: a systematic review of psychometric properties and clinical utility. J Rehabil Med. (2015) 47:193–203. 10.2340/16501977-1930 25590458

